# African and Asian Medicinal Plants as a Repository for Prospective Antiviral Metabolites Against HIV-1 and SARS CoV-2: A Mini Review

**DOI:** 10.3389/fphar.2021.703837

**Published:** 2021-08-25

**Authors:** Godwin Anywar, Muhammad Akram, Muhammad Amjad Chishti

**Affiliations:** ^1^ Department of Plant Sciences, Microbiology and Biotechnology, College of Natural Sciences, Makerere University, Kampala, Uganda; ^2^ Department of Eastern Medicine, Government College University Faisalabad, Faisalabad, Pakistan; ^3^ Department of Basic Clinical Sciences, Faculty of Eastern Medicine, Hamdard University, Karachi, Pakistan

**Keywords:** medicinal plants, antiviral, COVID-19, HIV/aids, immune system, infectious diseases

## Abstract

**Introduction:** The worldwide burden of viral infections has triggered a resurgence in the search for new and more efficient antiviral drugs. Scientists are also repurposing existing natural compounds such as the antimalarial drug artemisinin from *Artemesia annua* L. as potential drug candidates for some of the emerging and re-emerging viral infections such as covid-19

**Aim:** The aim of this review was to analyse the existing literature to explore the actual or potential natural antiviral compounds from African and Asian medicinal plants as lead compounds in the drug discovery process.

**Methods:** We searched the literature on African and Asian medicinal plant species as antiviral agents for HIV-1 and the novel coronavirus (SARS-CoV-2) in various databases and search engines such as Web of Science, Google Scholar and PubMed. The search was limited to *in vitro*, *in vivo*, and clinical studies and excluded *in silico* studies.

**Results:** We present 16 plant species with actual or potential antiviral activity against HIV-1 and SARS-CoV-2. These plant species span the continents of Africa and Asia where they are widely used for treating several other ailments.

**Conclusion:** Natural compounds from plants can play a significant role in the clinical management of HIV/AIDS and the covid-19 pandemic. More research needs to be conducted to investigate the potential toxicities of the various compounds and their efficacies in clinical settings.

## Introduction

The emergence and re-emergence of new infectious diseases especially of viral origin are of great concern to global public health (Louten, 2016; Mourya et al., 2019; Abrahão and de Arruda, 2020). This has reignited the search for natural compounds especially, from plants as potential sources of new drug candidates to add to the current armamentarium (Jans et al., 2020). This review focusses on HIV-1 and SARS-CoV-2, the viruses that cause AIDs and covid-19 respectively because over the last 50 years, these two viruses have caused huge pandemics globally: the HIV/AIDS pandemic and covid-19 pandemic (Illanes-Álvarez, et al., 2021). Since the start of the HIV/AIDS pandemic in the early 80’s, about 77.5 million people have become infected with HIV and over 34.7 million have succumbed to the diseases (UNAIDS, 2021). For the more recent covid-19 pandemic which begun in 2019, there have been 206,958,371 confirmed cases as of August 18, 2021 globally with 4,361,996 confirmed deaths, as of July 28, 2021 (WHO, 2021). The novel SARS-CoV-2 virus causes severe acute and fatal respiratory syndrome after gaining access to the host cells through the angiotensin-converting enzyme 2 (ACE2) co-receptors for the virus. The virus can also damage vital body organs by causing a cytokine storm (Ding et al., 2003; Hashemifesharaki and Gharibzahedi, 2020; Xu et al., 2020). Another similarity both pandemics share is causation by RNA viruses which have animal reservoirs and infections characterized by the increased synthesis of proinflammatory cytokines (Illanes-Álvarez et al., 2021).

Medicinal plants have a long history of healing many diseases. Natural compounds from plants are a rich reservoir of bioactive antiviral compounds. Recent scientific and technological advances have made it possible to easily isolate compounds from such plants with a wide range of biological activities (Martin and Ernst, 2003). Some natural products from plants contain metabolites that are key in preventing virus replication without affecting host metabolism. Such compounds are ideal candidates for the development of antiviral medicinal products with limited side effects. For instance, compounds such as 3β-friedelanol, friedelin, and epitaraxerol isolated from the ethanolic extract of *Euphorbia neriifolia* had potent anti-human coronavirus activity on human fibroblasts (MRC-5) infected cells (Chang et al., 2012). These natural plant products may additionally modify or enhance host immune responses against viruses; thus, alleviating symptoms and reducing mortality of viral infections (Kurokawa et al., 2010).

Fewer antiviral drugs are considered safe for human use than antibacterial agents mainly because viral pathogenesis is not as well understood at molecular level. Some viruses multiply and induce host cell lysis, while viruses incorporate themselves into the host chromosome and may remain dormant for years. Numerous viruses survive and thrive by hijacking the machinery of the host cell which includes different enzyme systems. Therefore, their inhibition is key to controlling viral diseases (Kurokawa et al., 2010).

Another approach that is vigorously being pursued is the repurposing of existing compounds and exploring their synergistic activity as potential drug candidates for diseases such as covid-19 (Prasad et al., 2020). Such compounds are often considered cheaper, safer, and efficacious alternatives to some of the currently available conventional drugs (Perera and Efferth, 2012). For instance, *A. annua* tea infusion is highly active against HIV-1 50% inhibitory concentration (IC_50_ = 2.0 μg/ml) but was originally used for treating malaria and fevers. Artemisinin, the main component from *A. annua* is currently the main stay for malaria treatment (Lubbe et al., 2012). Other studies are investigating the potential of already known compounds as potential candidates against SARS-CoV 2 and HIV-1. This mini-review reports on medicinal plant species and their metabolites as actual or potential antiviral candidates for HIV/AIDS and covid-19.

## Methods

We searched the literature on medicinal plant species used in Africa and Asia with scientific evidence of antiviral activity against either HIV-1 or SARS-CoV-2 or both in various databases and search engines such as Web of Science, Google Scholar and PubMed. We selected the most important plant species basing on scientific evidence from one or more studies confirming their antiviral activities. The search was limited to *in vitro*, *in vivo*, and clinical studies but excluded *in silico* studies. The search focused on English only studies conducted within the last 50 years. The selected plant species are presented in detail as follows:

## Results and Discussion

All the plant species selected are used in traditional medicine for treating various diseases including viral infections. Of the 16 medicinal plant species analysed, 11 were considered major because they had a larger body of scientific evidence to support their bioactivity or efficacy than the rest which were categoried as “other medicinal plant species with limited studies.”Table 1 shows a detailed breakdown of ten plant species with proven activity against SARS-CoV-2 and or HIV-1.

**TABLE 1:** Medicinal plant species with HIV-1 and SARS-CoV-2 activities.

**Plant name**	**Extraction solvent/Compound extracted**	**Antiviral activity and type of virus/strain/viral enzyme**		**Test model**	**Toxicity**	**Standard drug and potency/Control**	**Mechanism of action**	**References**
		**HIV-1**	**SARS-CoV-2**					
1*. Acacia catechu*	a) n-butanol fraction	a) IC_50_ = 12.9 μg/ml against Viral protease	ND	Enzymatic: (HIV-1 reverse transcriptase, integrase, protease, pro-viral genome integration and viral Tat protein mediated transactivation)	No cytotoxicity observed up to 100 μg/ml (CC_50_) using HIV_NL4.3_ in TZM-bl cells-based assay	Saquinavir (1 μM) 100% inhibition of HIV-1 PI	Inhibition of HIV-1 protease and interaction of viral Tat protein	Nutan et al. (2013)
	b) ethanol	b) IC_50_ = 3.6 ± 0.31 μg/ml		Cellular: HIV-1_NL4.3_ HIV-1_BaL_(R-5-tropic)				
	c) Water	IC_50_ = 1.8 ± 0.18 μg/ml		Cell-free virus-based assay using TZM-bl cells				
2. *Allium sativum*	Lectins (ASA and ASA1)	HIV-1	SARS-CoV Frankfurt 1 strain (CC_50_ > 100)	Vero E6 cells ATCC	IC_50_ > 100 μg/ml	Blank (infected cells incubated with medium)	Inhibits viral-cell attachment and viral reverse transcriptase in HIV-1 and infectious virus cycle of SARS- CoV-2	Tatarintsev et al. (1992), Keyaerts et al. (2007)
	Ajoene	(HIV)-1 (IIIB) EC_50 = _0.35 μM	ND	MOLT-4 cells	IC_50_ = 1.88 μM	Dextran sulfate	Blocks HIV-1 virus-cell attachment and adsorption	Walder, et al. (1997)
3. *Ancistrocladus korupensis*	Michellamine B	HIV-1 and 2	ND	Cultured human T-lymphoblastoid cells (CEM-SS and MT-2),	ND	Uninfected, non-drug- treated cells	Inhibits replication of both HIV-1 and HIV-2, and their CPE	Manfredi et al. (1991)
		IC_50_ = 18 μg/ml						
	Korundamine A	EC_50_ = 2 μM		CEM-SS and MT-2			Anticytopathic activity against HIV-1	Hallock et al. (1998)
		EC_50_ = 8, l0 and 6 μM		Several resistant strains of HIV			ND	
				CEM-SS/OClOO				
				MT2/A17				
				MT2/G9106 host cell/virus strain combinations, respectively				
4. *Artemisia annua*	Ethanol leaf extract	ND	EC_50_ = 34.5 μg/ml, SI = 31	Cellular: Vero E6 cells	ND	Absorbance of uninfected cell control (100%)	Inhibits effects on virus-induced CPE in a dose dependent manner	Li et al. (2005)
			EC_50_ = 39.2 μg/ml, SI = 27	SARS-CoV-1 BJ-001 strain				
			CC_50_ = 1,053.0 (±92.8) for both	SARS-CoV-1 BJ-006 strain				
	Water infusion	IC_50_ = 2.0 μg/ml)	ND	Cellular: HIV−1	No cellular toxicity on human cells with iFIGS- and deCIPhR systems	Efavirenz (a non-nucleosidic RT inhibitor in clinical use)	ND	Lubbe et al. (2012)
5*. Cullen corylifolium*	Ethanolic seed extract	ND	IC_50_ = 15 μg/ml	Enzymatic: SARS-CoV- papain-like protease (PLpro)	ND	Untreated	ND	Kim et al. (2014)
	6 isolated bioactive flavonoids		IC_50_ = 4.2–38.4 µM)	Cellular			Dose-dependent inhibition of PLpro	
6. *Euphorbia neriifolia*	Twenty-two triterpenoids and 1 flavonoid glycoside; 3β-friedelanol, 3β- acetoxy friedelane, friedelin, and epitaraxerol	ND	ND	Cellular in MRC-5 cells (20 μL, strain 229E in the XTT assay	ND	Actinomycin D (0.02 μg/mL)	Structure-activity relationship against HCoV	Chang et. al,( 2012)
7. *Mangifera indica*	Mangiferin	HIV-1_ⅢB_	ND	Cellular	Low cytotoxicity on C8166, MT-4, chronically-infected H9 cells and PBMC (CC_50_ > 1,000 μg/ml (2,369.67 μM)	IDV showed cytotoxicity on C8166 (CC_50_ value >500 μM)	Inhibits HIV-1ⅢB induced syncytium formation and inhibits HIV-1 protease	Wang et al. (2011)
		EC_50_ = 16.90 μM		Enzymatic				
		SI > 140						
		MT-2 cells		MT-2 cell line	Not toxic (IC_50_ = 125 μ/ml)	Zidovuline (IC_50_ = 3.5 μ/ml)	Reduces the CPE in MT-2 cells and prevented cell death	Guha et al. (1996)
		CC_50_ = 3.95 μ/ml						
		IC_50_ = 125 μ/ml						
8. *Panax ginseng*	Six 300 mg/capsules taken thrice daily	HIV-1	ND	*In vivo:* HIV-1-infected patients	ND	People without HIV-1 infection	Slows depletion of CD4 T cells and serum CD8 levels	Sung et al. (2005), Cho and Kim, (2017)
	Ginsenoside-Rb1 (100 μM)			Cellular			Prevents viral entry	Shehzad et al. (2012)
9*. Pelargonium sidoides*	Aqueous root extract	HIV-1 SI ≈ 45	ND	HIV-1 (various X4 and R5 tropic strains, and clinical isolates)	Low cytotoxicity: (CC_50_ ≤ 250 mg/ml)	Cells without virus inoculum (background) and cells exposed to virus particles in the absence of inhibitory compounds (attachment control)	Directly interferes with viral infectivity and blocks viral attachment and entry of HIV-1 particles to target cells	Helfer et al. (2014)
	Polyphenols	SI = 24	ND	ND	Low cytotoxicity: (CC_50_ ≤ 1,200 mg/ml)	ND	ND	ND
10. *Phyllanthus amarus*	Aqueous or alcohol extract	IC_50_ = 0.48–0.16 μg/ml	ND	Enzymatic HIV integrase	ND	Uninfected cells (negative control); untreated cells and a neutralizing serum (1:100 diluted) -positive control	Blocks HIV-1 attachment to cells and viral enzymes	Notka et al. (2004)
		IC_50_ = 8.17–2.53 μg/ml		Reverse transcriptase				
		IC_50_ = 21.80–6.28 μg/ml		Protease				
		IC_50_ = 2.65 μg/ml		Cellular: CD4-gp120 binding			Blocked interaction of HIV-1 gp120 and the CD4 receptor	
	Geraniin	IC_50_ = 0.48 μg/ml		Cellular: CD4-gp120 binding				
11. *Theobroma cacao*	Sodium hydroxide cocoa husk extract	HIV-1 SI = 311, 46	ND	HTLV-1-transformed T-cell lines MT-2 and MT-4	Cacao pigment was cytotoxic above 500 μg/ml to MT-2 and MT-4 cells	MOLT-4 cell and MOLT-4 cells treated with 250 μg/ml CP	Inhibits CPE of HIV-1 against HTLV-1-transformed T-cell lines MT-2 and MT-4 Inhibits syncytium formation between HIV-infected and uninfected lymphoblastoid T-cell line, MOLT-4	Unten et al., 1991; Sakagami et al., (2011)
	Cacao lignin-carbohydrate complex (LCC)	HIV-1	ND	ND			Inhibits antiviral and macrophage stimulatory activity	Sakagami et al. (2011)

CPE = Cytopathic effect; ND = Not Determined; SI = Selectivity Index.

### *Acacia catechu* (L.f.) Willd. Family: Fabaceae

*A. catechu* is a moderate size deciduous tree with a dark grayish or brown rough bark (Chopra et al., 1956). It is indigenous to India and other Asian countries, as well as East Africa (Patel et al., 2009; Sunil et al., 2019). The stem bark, aqueous and ethanol extracts of *A. catechu* caused the suppression of HIV-1 infection partly through their inhibitory effect on HIV-1 protease and partly due to the interference in interaction of viral Trans-Activator of Transcription (Tat) protein. The n-butanol fraction of *A. catechu* exhibited strong inhibitory activity against HIV proteases (IC_50_ = 12.9 μg/ml). *A. catechu* acts through various other mechanisms such as inhibition of fusion of infected cells and blocking RNA synthesis (Nutan et al., 2013). The n-butanol fraction showed a dose-dependent inhibition against HIV-1_NL4.3_ infection of peripheral blood mononuclear cells (PBMC), and against HIV-1_BaL_(R-5-tropic) as well as two different primary viral isolates of HIV-1 (Nutan et al., 2013). *A. catechu* contains various compounds such as kaempferol, quercetin, catechin, rutin, isorhamnetin, epicatechin, afzelechin, epiafzelechin, mesquitol, ophioglonin, aromadendrin and phenols (Li et al., 2010). However, the specific bioactive compounds responsible for the anti-HIV activity were not established.

### *Allium sativum* L. Family, Amaryllidaceae

*A. sativum* (garlic) is a member of the onion family. It is a hardy perennial herb likely to have originated in central Asia where it was used as a food and medicine. Traditionally, it was used for preventing infections and the treatment of colds, influenza, bronchitis, whooping cough, gastroenteritis, dysentery and skin problems (Rivlin, 2001; Lissiman et al., 2014) and to boost immunity in people living with HIV/AIDS (Anywar et al., 2020a). *A. sativum* has a range of biological activities, including antiviral properties. It inhibits viral-cell attachment, viral reverse transcriptase, and further destruction of CD4^T^ cells (Tatarintsev et al., 1992; Walder et al., 1997). Ajoene, a compound isolated from garlic showed dose-dependent HIV inhibition (EC_50_ = 0.35 μM). The lectins (ASA and ASA1) from garlic inhibited both HIV-1 replication and viral attachment in the later stages of infectious virus cycle of SARS-CoV (Keyaerts et al., 2007). Garlic extract inhibited angiotensin converting enzyme (ACE)2, a functional receptor for SARS-CoV Li et al. (2003) by 39.57% at a dose of 10 mg/ml Chaudhary et al. (2020) indicating its potential antiviral activity against SARS-CoV. *A. sativum* contains various other bioactive compounds including alliin, allicin, vinyldithis and flavonoids (Chavanet al., 2016; Kim et al., 2018; Baek et al., 2019; Phan et al., 2019; El-Saber Batiha et al., 2020).

### *Ancistrocladus korupensis* D.W.Thomas and Gereau Family: Ancistrocladaceae

*A. korupensis* is a West African liana with the only known population limited within Korup National Park and its vicinity, in Cameroon. *A. korupensis* has antiviral activity against HIV-1 and HIV-2. Different compounds with anti-HIV activity have been isolated from its bark and leaves such as michellamine B Manfredi et al. (1991), Foster and Sork (1997) and korundamine A (Hallock et al., 1998). Michellamine B is a naturally occurring naphthylisoquinoline alkaloid that inhibits the replication of both HIV-1 and HIV-2, and their associated cytopathic effects (CPE) on cultured human T-lymphoblastoid cells (CEM-SS and MT-2), with a 50% effective concentration (CC_50_) near 18 μg/ml (Manfredi et al., 1991). Korundamine A is also a naphthylisoquinoline alkaloid which is heterodimeric. Korundamine A exhibited anticytopathic activity against HIV-1 (EC_50_ = 2 μM) (Hallock et al., 1998)*.* The anti-HIV activity of korundamine A is comparable to the anti-HIV activity of the michellamines. Korundamine A is also active against several resistant strains of HIV, with EC_50_ values of 8, l0 and 6 μM for the CEM-SS/OClOO, MT2/A17 and MT2/G9106 host cell/virus strain combinations, respectively (Hallock et al., 1998)*.* Five michellamine-type dimeric naphthylisoquinoline alkaloids: michellamines A_2_, A_3_, A_4_, B_2_, and B_3_, were isolated from the root bark of a related species, the Central African liana *Ancistrocladus congolensis*, along with their two known parent compounds, the michellamines A and B (Bringmann et al., 2016)*.*

### *Artemisia annua* L., Family Asteraceae

*A. annua* is a shrub, often growing over 2 m high (Ferreira et al., 1997). It is native to China and has traditionally been used to treat malaria and fevers Willcox et al. (2004), Anywar et al. (2020b) and to boost immunity in people living with HIV/AIDS (Anywar et al., 2020a). *A. annua* extracts inhibit various viruses (Efferth et al., 2008). The ethanol extracts of *A. annua* significantly inhibited the effects on virus-induced CPE in SARS-CoV. The ethanol leaf extract of *A. annua* had antiviral activity against SARS-CoV-1 BJ-001 strain (EC_50_ = 34.5) and BJ-006 strain (EC_50_ = 39.2). *A. annua* was proposed as one of the potential candidates for the development of new anti-SARS-CoV drug (Li et al., 2005). In addition, A. annua tea infusion is highly active against HIV (IC_50_ = 2.0 μg/ml) but the main component, artemisinin was inactive at 25 μg/ml (Lubbe et al., 2012). The specific phytochemical compounds responsible for its anti-HIV-1 and anti-SARS-CoV activity have not established although it contains bioactive compounds such as quercetin, polyphenols, saponins, sterols, dicaffeoylquinic acid polyaccharides (Lin et al., 2014).

### *Cullen corylifolium* (L.) Medik. Syn *Psoralea corylifolia*, Family: Fabaceae

*C. corylifolium* Syn: *P. corylifolia* is a is a popular traditional medicinal herb that occurs mainly in Indonesia, Malaysia, Bangladesh, India, China, and Sri Lanka. *P. corylifolia* has well known antiviral, antioxidant, antibacterial and antidepressant activities (Chopra et al., 2013; Alam et al., 2018). *P. corylifolia* seed extract exhibited high activity against SARS-CoV-papain-like protease (PLpro) (IC_50_ = 15 μg/ml). SARS-CoV Plpro is an important enzyme in SARS virus replication (Kim et al., 2014). *P. corylifolia* contains various bioactive components (Schneiderová et al., 2013; Kim et al., 2014). All the six isolated bioactive flavonoids from *P. corylifolia*: bavachinin, neobavaisoflavone, isobavachalcone, 4′-O-methylbavachalcone, psoralidin and corylifol produced a dose-dependent inhibition of PLpro (IC_50_ = 4.2–38.4 µM) (Kim et al., 2014).

### *Euphorbia neriifolia* L Family: Euphorbiaceae

*E. neriifolia* is a spiny herb native to Southeast Asia with a native range from Iran to Myanmar (Hieh et al., 1993; POWO, 2020). The herb is currently cultivated in southern Taiwan (Hieh et al., 1993). Terpenoids isolated from *E. neriifolia* exhibited a structure-activity relationship against the human coronavirus (HCoV). Twenty-three compounds (22 triterpenoids and 1 flavonoid glycoside) have been isolated from the ethanolic leaf extract of *E. neriifolia* leaves. Specifically, 3β-friedelanol exhibited more potent anti-viral activity against the corona virus (HCoV, strain 229E) than the positive control, actinomycin D (0.02 μg/ml) Chang et al. (2012) in MRC-5 cells (human fibroblasts cells).

### *Mangifera indica* L Family: Anacardiaceae

*M. indica* is a medicinal and fruit tree widely used in the traditional medicine in India, and Africa (Makare et al., 2001; Anywar et al., 2020a). *M. indica* possess antiviral activities which have been attributed to the compound mangiferin, a naturally occurring gucosylxanthone (Guha et al., 1996; Wang et al., 2011). Mangiferin inhibited HIV-1_ⅢB_ induced syncytium formation at non-cytotoxic concentrations, with a 50% effective concentration (EC_50_) of 16.9 μM. Mangiferin also demonstrated antiviral activity against various laboratory-derived, clinically isolated and resistant HIV-1 strains of HIV. Mangiferin acts by inhibiting HIV proteases (Wang et al., 2011). Mangiferin reduced the CPE in MT-2 cells and prevented cell death at > 10 μ/ml with a CC_50_ of 3.95 μ/ml and a lower IC_50_ of 125 μ/ml than the standard drug zidovuline (IC_50_ of 3.5 μ/ml). Mangiferin was thus not toxic at such high concentrations compared to the standard drug (Guha et al., 1996).

### *Panax ginseng* C.A.Mey. Family: Araliaceae

*Panax ginseng* is a highly valued medicinal plant that has been used for thousands of years. It is mostly cultivated in China, Korea, and Japan (Shehzad et al., 2012; 2013; Bai et al., 2018). Ginseng is a perennial herb whose rhizome is an important determinant of its quality (Choi, 2008). The consumption of Korean ginseng was shown to slow the depletion of CD4 T cells and serum CD8 levels in HIV-1-infected patients (Sung et al., 2005). A retrospective study that analyzed 252 HIV-1 patients diagnosed from 1986 to 2013 prior to the initiation of antiretroviral therapy showed that *P. ginseng* prolonged survival in HIV-1 patients. The study also showed significant correlations between the total amount of *P. ginseng* consumed and survival time (*r* = 0.64, *p* < 0.0001) and between total amount of *P. ginseng* and mean annual decrease in CD4^+^ T-cell count in all 252 patients (*r* = −0.17, *p* < 0.01) (Cho and Kim, 2017). The ginsenoside-Rb1, which is one of the active components of *P. ginseng* (Shehzad et al., 2012), showed antiviral activity against FP-21399, a bis-azo derivative with HIV inhibition activity by preventing viral entry at 100 μM (Zhang et al., 1998). The major pharmacologically active components of ginseng root are ginsenosides which make up 2–3% of the root (Jeong et al., 2003; Shehzad et al., 2012).

### *Pelargonium sidoides* DC. Family: Geraniaceae

*P. sidoides* is a popular herb whose roots are widely used in traditional medicine in South Africa for different ailments such as diarrhoea, dysentery, ear, nose, throat disorders and respiratory tract infections (Kolodziej, 2002; Brendler and van Wyk, 2008; Michaelis et al., 2011). *P. sidoides* has potent anti-HIV-1 activity. It protected PBMC and macrophages from infection with various X4 and R5 tropic HIV-1 strains, and clinical isolates by directly interfering with viral infectivity and blocking viral attachment and entry of HIV-1 particles to target cells. *P. sidoides* contains active polyphenolic compounds with low cytotoxicity (Helfer et al., 2014). However, the specific phytochemical compounds responsible for its anti-HIV-1 activity were not established.

### *Phyllanthus amarus* Schumach. and Thonn., Family: Phyllanthaceae

*Phyllanthus amarus* (Syn *P. niruri*) is a small shrub that is widespread in the tropics. The aqueous or alcohol extracts of *P. amarus* extracts inhibited HIV replication both *in vivo* and *in vitro* by blocking HIV-1 attachment to cells. *P. amarus* also inhibited the HIV-1 enzymes: integrase by 0.48–0.16 μg/ml, reverse transcriptase by 8.17–2.53 μg/ml, and protease by 21.80–6.28 μg/ml. The extracts blocked the interaction of HIV-1 gp120 and the CD4 receptor with 50% inhibitory concentrations of 2.65 μg/ml for the aqueous/alcohol extract and 0.48 μg/ml for geraniin, an isolated egallotanin from the plant. Additionally, sera from human volunteers reduced HIV replication by more than 30% at a concentration of 5% *in vivo* (Notka et al., 2004). The alkaloidal extract of *P. niruri* selectively inhibited the cytopathic effects of HIV-1 on human MT-4 cells at the tested concentrations (CC_50_ = 279.85 μg/ml; EC_50_ = 20.98 μg/ml). The selectivity index (SI) (defined as CC_50_/EC_50_) of the extract for the viral cells was 13.34 (Naik & Juvekar, 2003). *P. amarus* contains gallotannins, and the ellagitannins: geraniin and corilagin were shown to be the most potent mediators of the anti HIV-1 activities (Notka et al., 2004).

### *Theobroma cacao* L., Family: Malvaceae

*T. Cacao* is a small evergreen tree native to South America (Baliga et al., 2014). The seeds of *T. cacao* are often used in the food industry especially for chocolate making (Wickramasuriya and Dunwell, 2018). *T. cacao* has anti-HIV activity. A sodium hydroxide cocoa husk extract inhibited the CPE of HIV-1 against HTLV-1-transformed T-cell lines; MT-2 and MT-4. Furthermore, the extract also inhibited syncytium formation between HIV-infected and uninfected lymphoblastoid T-cell line, MOLT-4. The extract is thought to act by inhibiting the adsorption of the virus (Unten et al., 1991). Cacao lignin-carbohydrate complex (LCC) has also shown strong antiviral and macrophage stimulatory activity (Sakagami, et al., 2010). However, cacao mass LCC demonstrated higher anti-HIV activity than cacao husk (Sakagami et al., 2011). The husk extract is made up of condensed or polymerized flavonoids such as catechin and anthocyanidin which are sometimes complexed with glucose (Kimura, 1979). *T. cacao* contains several bioactive compounds such theobromine, and polyphenols (Goya et al., 2016; Oyeleke, et al., 2018). However, the specific phytochemical compounds responsible for its anti-HIV-1 activity were not established.

### Other Medicinal Plant Species With Limited Studies

Other plant species with promising as antiviral but with limited research are presented below.

#### *Lindera aggregata* (Sims) Kosterm. Family: Lauraceae

*L. aggregata* is a common herb in China and Japan. Its roots are mainly utilized for the treatment of pain, inflammation, indigestion, cold and hernia (Xiao et al., 2011; Wei et al., 2017). L. aggregata exhibited significant antiviral activity by inhibiting virus-induced CPE against the SARS-CoV strain BJ001 (EC_50_ = 88.2 ± 7.7 μg/ml) (Li et al., 2005). The specific phytochemical compounds responsible for its anti-SARS-CoV-2 activity were not established.

#### *Lycoris radiata* (L'Hér.) Family; Amaryllidaceae

*L. radiata* is a herb native to China, Korea, Japan, and Nepal with various biological activities including: antiviral, and anti-inflammatory (Cedrón et al., 2010; Lamoral-Theys et al., 2010; Kretzing et al., 2011). The ethanolic stem extract of *L. radiata* exhibited inhibitory effects against SARS- CoV-2, which have been attributed to lycorine Li et al. (2005), the main active component (Yang et al., 2019).

#### *Onopordum acanthium* L. Family: Asteraceae

*O. acanthium* is a herbaceous plant native to Europe, Xinjiang and W. Himalaya in Asia, and NW. Africa (POWO, 2020). *O. acanthium* has anti-inflammatory, anti-cancer, antiviral, and antioxidant properties. *O. acanthium* was effective at inhibiting ACE-2 activity by more 80% (Sharifi et al., 2013), which could potentially be effective against SARS-CoV-2. Although the specific compounds responsible for its anti-SARS-CoV-2 activity were not established, extracts of *O. acanthium* have a wide range of bio-active components including flavonoids, triterpenoids, phenylpropanoids, sesquiterpene lactones and sterols (Csupor-Löffler et al., 2014; Abusamra et al., 2015).

#### *Pyrrosia lingua* (Thunb.) Farw., Family: Polypodiaceae

*P. lingua* is an epiphytic fern that mainly occurs in Korea, Japan, China, and other Asian states. *P. lingua* has antiviral, antioxidant, antibacterial and anti-cancer activities (Zheng, 1990; Fan et al., 2020). The leaves of *P. lingua* have traditionally been used to treat various viral infections. The chloroform leaf extract of *P. lingua* had antiviral activity against SARS-CoV-1 BJ-001 strain (EC_50_ = 43.2) and BJ-006 strain (EC_50_ = 40.5). *P. lingua* was proposed as one of the potential candidates for the development of new anti-SARS-CoV drugs (Li et al., 2005). Although the specific compounds responsible for its anti SARS-CoV activity were not established, *P. lingua* is known to contain several bioactive compounds such as flavonoids, chlorogenic acid, mangiferin, isomangiferin, astragaline and trifoline (Xiao et al., 2017). In separate studies, mangiferin has been shown to have potent anti-HIV-1 activities (Guha et al., 1996; Wang et al., 2011) as discussed under *M. indica*.

#### *Trichosanthes Kirilowii* Maxim., Family Cucurbitaceae

*T. kirilowii* is a Chinese medicinal herb with anti-HIV-1 activity (Lo et al., 2017). Trichobitacin from *T. kirilowii* suppresses HIV-1 induced formation of cell syncytia. Trichobitacin is a novel ribosome-inactivating protein that has been isolated from the roots *T. kirilowii* (Zheng et al., 2000). Trichobitacin also reduced the expression of HIV-1 p24 antigen and the number of HIV antigen positive cells in acutely HIV-1 infected culture (IC_50_ = 5 μg) (Zheng et al., 2000). According to Byers et al. (1994), clinical studies involving the use of trichosanthin to treat AIDS patients failing treatment with antiretroviral agents such as zidovudine may help to prevent loss of CD4^+^ cells.

## Conclusion

This review shows that 16 plant species used traditionally in Africa and Asia for treating various ailments possess *in vitro* and *in vivo* anti-HIV-1 and anti-SARS-CoV-2 activity. This affirms the potential of medicinal plants as a reservoir of potential antiviral compounds. These compounds need further investigation as potential drug candidates for HIV-1, SARS-CoV-1 and other pathogenic agents. Most of the plant species lack clinical studies to support their efficacy despite showing *in vitro* bioactivity*.* The potential therapeutic effects of these medicinal plants and others against SARS-CoV-2 and HIV-1 should be explored through further research, on their efficacy and safety, herbal-drug interactions, clinical trials and product development.

Only two plant species; *A. sativum* and *A. annua* showed biological activity against both HIV-1 and SARS-CoV-2. Eight of the 16 plant species plant had been tested for activity against SARS-CoV-2 whereas 10 had been tested against HIV-1. Only *P. ginseng* and *T. kirilowii* were subjected clinical trials. The rest of the plant species had only been evaluated in cellular based-assays *in vivo.*

Although the general phytochemistry of the plant species reviewed is known, it is only in a handful of cases where the specific compounds responsible for the antiviral activity was determined. The assays conducted involved the use of various extracts including crude and pure compounds as well as fractions of the extracts. The commonest extracts or solvents used were ethanol and water. The isolated pure compounds tested were geraniin from *P. amarus*, ginsenoside-Rb1 from *P. ginseng,* mangiferin from *M. indica,* six isolated bioactive flavonoids from *C. corylifolium*, michellamine B and korundamine A from *Ancistrocladus korupensis* and lectins and ajoene from *A. sativum*.

The cytotoxicity of most of the plant species was generally low where determined. However, the cytotoxicity data need to be backed by *in vitro* or *in vivo* toxicity data*.* This is important because be toxic and need to be thoroughly investigated to rule out or mitigate any possible harmful effects before use (Anywar et al., 2021). Medicinal plants could potentially be toxic and need to be thoroughly investigated to rule out or mitigate any possible harmful effects before use. This can be seen from the compound trichosanthin from *T. kirilowii* used to treat AIDS patients. Even though clinical studies involving the use of trichosanthin to treat AIDS patients failing treatment with antiretroviral drugs such as zidovudine may help to prevent loss of CD4^+^ cells (Byers et al. (1994). Trichosanthin has undesirable side effects which have greatly restricted its clinical application (Zhao et al., 1999).

The plant extracts reviewed work mainly as inhibitors of the various viral enzymes involved in the virus life cycle. Although the mechanisms of action of most of the major medicinal plants were well understood, there were cases such as *Cullen corylifolium* against SARS-CoV-2, Korundamine A from *Ancistrocladus korupensis* in the different strains of HIV-1 and the water infusion *Artemisia annua* against HIV-1 where the mode of action was no determined.

Although the aspect of herb-drug interactions between the medicinal plants and conventional medicines are largely unknown, potential threats exist and need to be investigated. For instance, *P. ginseng* was found to interact with some anti-HIV drugs and change their pharmacokinetic properties. Ginsenoside Rh2, a compound from *P. ginseng* increased the accumulation and decreased the efflux of ritonavir through P-glycoprotein (P-gp) in Caco-2 cells and MDCK-MDR1 cells (Shi et al., 2013).

The potential therapeutic effects of *T. cacao* against SARS-CoV-2 should be explored through further research.
